# Nano-Based Co-Delivery System for Treatment of Rheumatoid Arthritis

**DOI:** 10.3390/molecules27185973

**Published:** 2022-09-14

**Authors:** Shixin Zhang, Miaomiao Zhang, Xiangyu Li, Ge Li, Bo Yang, Xinyue Lu, Yang Gao, Fengying Sun

**Affiliations:** 1 School of Life Sciences, Jilin University, Changchun 130012, China; 2 College of Education and Humanities, Suzhou Vocational University, Suzhou 215104, China

**Keywords:** rheumatoid arthritis (RA), co-delivery system, drug delivery, nanoparticles, phototherapy, combination therapy

## Abstract

A systemic autoimmune condition known as rheumatoid arthritis (RA) has a significant impact on patients’ quality of life. Given the complexity of RA’s biology, no single treatment can totally block the disease’s progression. The combined use of co-delivery regimens integrating various diverse mechanisms has been widely acknowledged as a way to make up for the drawbacks of single therapy. These days, co-delivery systems have been frequently utilized for co-treatment, getting over drug limitations, imaging of inflammatory areas, and inducing reactions. Various small molecules, nucleic acid drugs, and enzyme-like agents intended for co-delivery are frequently capable of producing the ability to require positive outcomes. In addition, the excellent response effect of phototherapeutic agents has led to their frequent use for delivery together with chemotherapeutics. In this review, we discuss different types of nano-based co-delivery systems and their advantages, limitations, and future directions. In addition, we review the prospects and predicted challenges for the combining of phototherapeutic agents with conventional drugs, hoping to provide some theoretical support for future in-depth studies of nano-based co-delivery systems and phototherapeutic agents.

## 1. Introduction

As a relatively common systemic autoimmune disease, rheumatoid arthritis (RA) causes severe mental and physical suffering in about 0.5–1% of the world’s population [1]. According to research, the age-standardized prevalence and incidence of RA are still rising [2], and it can potentially trigger other autoimmune diseases and raise the risk of cancer progression [3,4]. As a result, RA is now considered a major public health issue on a global scale. Although the pathogenesis of RA is not very clear so far, numerous researchers have demonstrated that inflammatory cells, inflammatory factors, and some metabolites are directly related to the development and progression of RA [5,6,7,8,9,10]. For instance, macrophages, neutrophils, lymphocytes, chondrocytes, fibroblasts, and other cells may cause irreversible damage and the degeneration of synovium and cartilage in joints under the activation of specific cytokines [11,12,13,14,15,16]. The severity and underlying mechanisms of RA are influenced by many pro-inflammatory substances and inflammatory cells [17,18,19,20]. Therefore, the complex pathogenesis makes various treatments for RA less effective, especially when using a single drug.

Nowadays, apart from invasive surgery, drugs, such as disease-modifying antirheumatic drugs (DMARDs) [21], non-steroidal anti-inflammatory drugs (NSAIDs) [22,23], and glucocorticoids (GCs) [24], are currently used for RA treatment. Despite the apparent therapeutic effects of these drugs, their drawbacks cannot be ignored. NSAIDs are often used as symptomatic drugs to relieve swelling and pain in RA but do not reverse the course of the disease; GCs can inhibit the function and proliferation of Th1 cells and thus reduce the production of pro-inflammatory factors, but slightly higher doses and long-term use may have adverse effects [25]; the efficacy of methotrexate (MTX), the most commonly used DMARDs, is unquestionable, but in recent years it has been found that the use of MTX may affect the human intestinal flora and thus the immune system [26,27,28]. In addition, nucleic acid-based drugs are also being used in studies to influence the RA disease process, such as small interfering RNA (siRNA) and small molecule RNA (microRNA, miRNA) interfering with the expression of inflammation-related proteins [29,30,31]. Although nucleic acid therapies offer diverse targets, excellent specificity, and little drug resistance, they also have issues that need to be resolved. For example, siRNAs are rapidly broken down by enzymes and are unstable in physiological settings [32]. Exogenous siRNA can also have off-target effects and immune clearing [33]. Accordingly, a single drug will not only be ineffective, but also show all of its flaws. Thus, co-delivery is becoming a more common topic of research [34].

**Figure 1:**
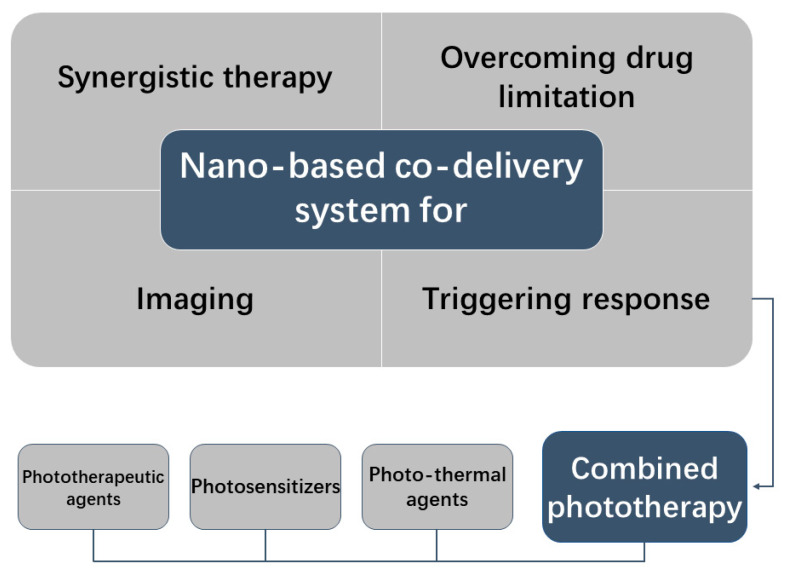
A brief graphical scheme of this review.

Since its inception to the present, nanotechnology has developed. A molecular milestone has been reached in the sciences. Thus, it is inevitable that nanotechnology will be widely applied. Nanotechnology is used in the nutraceuticals industry for a variety of reasons, including packaging, testing, and the progressive emergence of numerous nano additives and nano nutritious products [35]. In the environmental field, nanoplatforms can also be designed to detect the presence of harmful substances such as bacteria [36]. Nanotechnology also becomes the key component in the medical sector [37,38,39,40]. Nano delivery systems have made outstanding contributions to the treatment of various diseases. However, monotherapy with the delivery of only one drug is not good enough to achieve disease treatment. The nano-based co-delivery system came into being while solving these problems well. First, the co-delivery formulation can precisely transport the loaded drug into the target cells while lowering systemic toxicity by targeting inflammatory areas by surface modification with certain moieties. Meanwhile, it tends to reduce the release rate by encapsulating the drug, allowing for relatively large dose delivery and reducing side effects. Co-delivered biologics synergistically improve the efficacy of RA treatment and overcome drug limitations through different mechanisms. On this basis, the advantages of biological applications are further enhanced to achieve the greatest possible drug benefits. In some ways, it also optimizes responsive nano-based co-delivery formulations and advances a more individualized, non-invasive approach to treatment. According to these, we reviewed many experimental cases to explain the superiority of the co-delivery system and the shortcomings (Figure 1).

In this review, we discuss the strengths and weaknesses of different types of nano-based co-delivery systems from another perspective. We have focused on the common properties of co-delivery systems for different purposes and exploited the advantages of combined therapies instead of focusing on the nanoparticle dosage form. In particular, for the topical light-response triggered delivery systems, we discuss their commonalities and characteristics as part of the co-delivery system in details. Moreover, we also review the combined application prospects and predicted challenges of phototherapeutic agents with traditional drugs, which we hope will provide some theoretical support for future in-depth research on nano-based co-delivery systems and phototherapy agents.

## 2. Rationale for Combination Therapy

**Figure 2:**
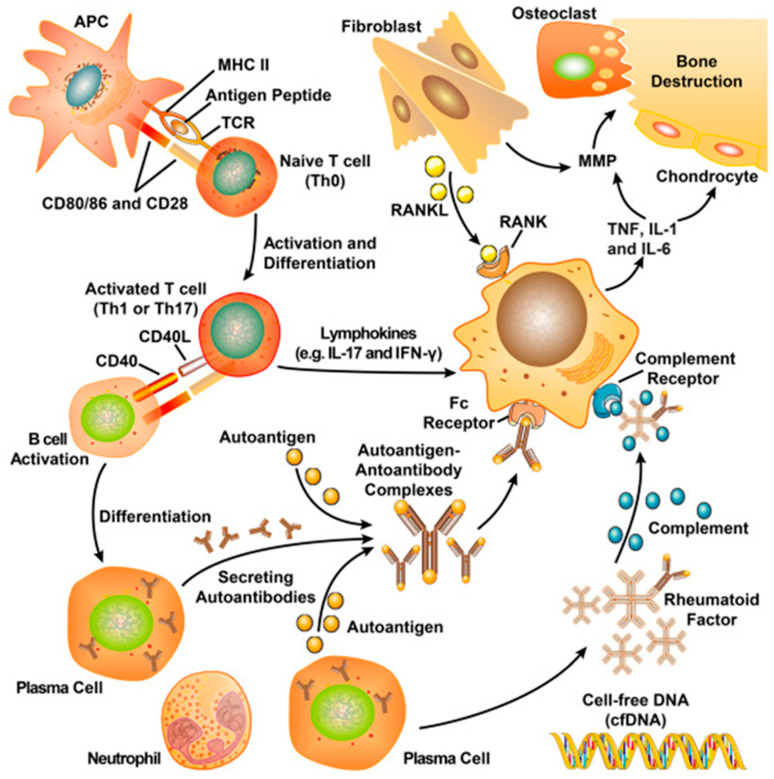
The complex pathogenesis of RA [5]. It includes different inflammatory factors produced by cells; matrix metalloproteinase and nuclear factor-κB signaling pathways; and activation of T cells. (Reprinted with permission from Ref. [5]. 2022, Wenjing Zhang).

Rheumatoid arthritis is an autoimmune illness that affects the entire body and is characterized by long-term, chronic bone degradation. We are aware of the complexity of the RA pathogenesis processes. The reasons for pathogenesis are not completely understood. They are related to a variety of factors, so we can hardly solve the problem by preventing the disease, but only by treating it after its onset to slow down the disease process. The pathophysiology of RA is thought to involve both genetic and environmental factors [41,42,43,44]. In contrast, during various phases of the RA disease process, synovial tissue macrophages, fibroblasts, and immune cells, such as T and B lymphocytes, also play a crucial role (Figure 2) [45]. Among them, macrophages, which can be stimulated to differentiate into different phenotypes, are widely used as targets to modulate the exertion of immune effects [46]. Drugs for the treatment of RA have emerged over the years, including chemical, biological, and some synthetic agents. However, monotherapy, targeting only one of these targets, often fails to completely inhibit damage to the joint site from other pathways. This is one of the justifications for delivering combination therapy concurrently. Combination therapy frequently suppresses the disease process through various pathways to treat or enhance a patient’s condition [47]. Additionally, the efficacy is more pronounced compared to single delivery [48,49].

**Figure 3:**
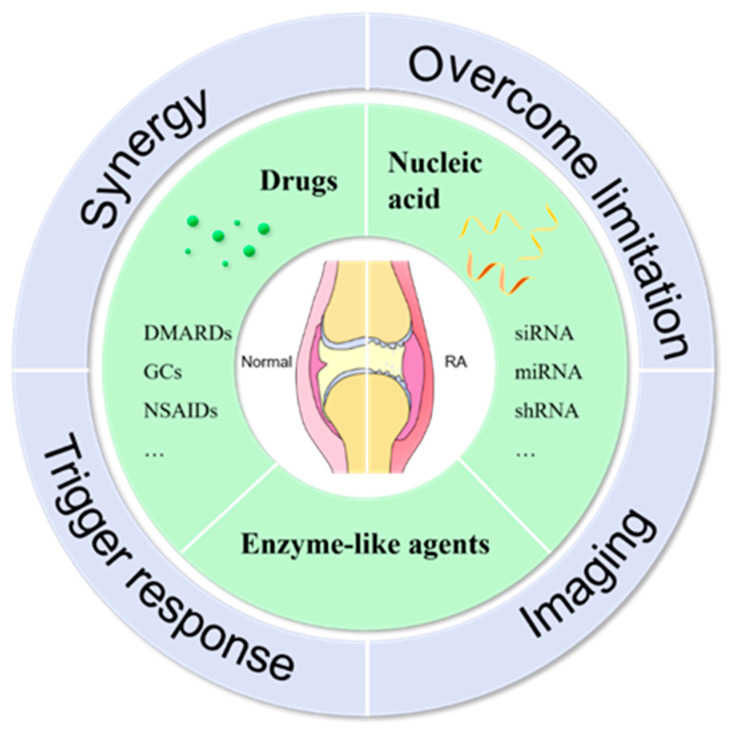
Commonly used drugs for the treatment of rheumatoid arthritis include: Small molecule drugs, nucleic acid drugs, and enzyme-like agents that co-deliver different drug mechanisms to relieve of inflammation, overcome the therapeutic limitations of individual therapies, enable imaging, and activate response molecules within the delivery system to trigger response-based therapy.

Despite the diversity of therapeutic drugs, they still have some fatal drawbacks. Resistance to drugs is common, especially in RA, a disease that requires long-term, chronic treatment. Drugs are not targeted to the joints, and these chemical drugs have a toxic solid effect, especially on the liver and kidney tissues. In addition, the prolonged use of conventional therapeutic treatments causes the body to become more resistant, which increases the need for additional drugs. However, as the quantity of pharmaceuticals increases, toxicity and resistance to the effect rise as well, creating a vicious cycle that worsens patient suffering. At the same time, imaging is also a problem to be solved if one wants to fully understand or monitor the disease process in real-time, or if the patient needs to achieve an informed heart through visual pictures. Ideally, both treatment and imaging outcomes would be accomplished after drug administration. As medical advances have gradually emerged, a variety of nanoparticles, nano micelles, and hydrogel carriers have been used to deliver therapeutic drugs, which increase the ability to target drugs to joints, reduce drug dosage and toxicity, and even add the ability to slow-release drugs and enhance patient compliance. One therapeutic effect, though, is still insufficient to address the many pathogenic pathways of RA. Trigger therapies that also deliver chemicals have therefore been developed to treat RA. As well as maintaining the therapeutic effects of the underlying medications, photodynamic and photothermal therapy improve the capacity to kill inflammatory cells and target the inflamed joints. The drug-carrying device can target the inflammatory location using a light response mechanism, and only near-infrared light is needed for long-term use to alleviate suffering. The synergistic effect of photoresponse and drug therapy dramatically reduces the amount of drugs used and its toxic effects, providing more effective treatment. Finally, based on some of the above characteristics of the disease itself and the cure, co-delivery is the superior choice and the rationale for co-delivery due to the advantages of less long-term toxicity, lower drug resistance, better targeting ability, and better patient suitability (Figure 3).

## 3. Nano-Based Co-Delivery System of Traditional Agents

### 3.1. Co-Delivery for Synergistic Therapy

The drugs commonly used for delivery in recent years are mainly DMARDs, NSAIDs, GCs, and other small molecule drugs for the treatment of RA by chemotherapy. Nucleic acids have also been successfully developed as therapeutic agents for RA through RNA interference technology in the last 20 years. In addition, enzymes can play a critical regulatory role at the site of inflammation [50]. Because they each control inflammatory conditions through different mechanisms resulting in greater cooperation between drugs than monotherapy, nano-based co-delivery system tends to take advantage of synergy. Meanwhile, it is mentioned that combination therapy is also indicated for the treatment of RA in the 2019 update of the European League Against Rheumatism (EULAR) guidelines [21]. Accordingly, the co-delivery synergistic strategy can be simply classified into different small molecule chemical drugs, chemical drugs with nucleic acid drugs, and chemical drugs and nanozymes with enzyme-like activity.

#### 3.1.1. Different Small Molecules

The first-line medication for the treatment of RA is MTX, one of the most well-known medications. In order to increase the therapeutic effectiveness of MTX, many trials typically co-delivered other medications [51]. Additionally, they display higher favorable treatment data. For instance, surface-modified hyaluronic acid targets the site of inflammation in polylactide–glycolic acid (PLGA) copolymer nanoparticles, loaded with MTX and radionuclides, which only extend drug release to 70% within 80 h [52]. The co-delivery of two therapeutic modalities, namely radiotherapy and chemotherapy, makes nanoparticles suitable for radio synovectomy and specific targeted antirheumatic therapy. Compared to exposure to a single drug, the effect of the dual mechanism on activated macrophages was better at 120 h. It is not difficult to see from the data that it is a synergistic effect. In vivo, microspheres modified with β-glucan were more effective in treating RA when MTX and vanillin were co-delivered than when either of these two drugs were used alone [53]. In addition, they were loaded 64.5% and 44.2%, respectively. This not only indicates that microspheres have substantially higher drug loading and encapsulation rates than conventional dosage forms, but also that the co-delivery of two small molecules enhances their mutual efficacy.

Of course, other traditional medicines have also been studied for co-delivery. A peptide vaccine carrying a multi-epitope citrullinated peptide (Cit-ME) and rapamycin (RAP) were loaded into the nanoparticles [54]. It can both drive the production of anti-inflammatory factors and upregulate regulatory T cells. This strategy promotes immune tolerance and improves RA tolerance in vivo by reducing the intensity of autoimmunity. Moreover, the gel, which further inhibits RA by reducing NO concentrations and inflammatory factor levels in the inflammatory environment, designed to deliver dexamethasone (DEX) and small molecule cross-linkers of NO response breaks [55].

Alternatively, it is also possible to load different small molecules into different nanocarriers separately. Even this approach can prevent some unwanted reactions between the two drug molecules. For instance, Zewail et al. encapsulated leflunomide (LEF) and DEX in nanoliposomes and PLGA nanoparticles separately and the two nanocarriers were subsequently put into a thermosensitive hydrogel [56]. Its superiority to monotherapy is also easily observed. There are many small-molecule drugs. In addition, there may be different degrees of synergy between any two. Every attempt by scholars has provided the possibility of co-delivery in the treatment of RA.

#### 3.1.2. Co-Delivery with Nucleic Acid Drugs

Researchers’ focus has recently been drawn to nucleic acid medications, due to their ability to molecularly control inflammation [57,58]. The main nucleic acid drugs currently used for the treatment of RA are small interfering RNA (siRNA), short hairpin RNA (shRNA), and small molecule RNA (miRNA). By pairing with the target messenger RNA (mRNA) and inducing mRNA degradation, their method of action prevents the expression of inflammatory components in the relevant cells. Among these, siRNA is frequently used as a drug to treat RA. RA synovial macrophages were transfected with Mcl-1 siRNA to reduce Mcl-1 expression, which induced apoptosis and slowed the expression of pro-inflammatory factors [59]. As a result, chitosan nanoparticles are encapsulated in acid-sensitive microspheres of PLGA and PCADK, while Mcl-1 siRNA are adsorbed on the nanoparticles [60]. Similarly, the mechanism of switching Mcl-1 siRNA to miR-124 is similar [49]. It is clear that polymeric nanoparticles can work alone or in concert with one another based on some of their inherent characteristics, rather than solely being dependent on the action of chemical reagents. We will not go into much detail though and will instead concentrate on the effect of the co-administration of chemical and nucleic acid drugs. Combination therapy was more effective than single treatment based on outcomes, such as scores, toe thickness, and proinflammatory factors. More typically, Li et al. used MSNs loaded with hypoxia-activated prodrugs followed by the adsorption of Mcl-1 siRNA via surface-modified positively charged PEI-FA to co-deliver chemotherapeutic and nucleic acid drugs, which not only killed activated macrophages but also inhibited the expression of inflammatory factors at the molecular level [61]. This method involved loading MSNs with hypoxia-activated prodrugs, followed by the adsorption of Mcl-1 siRNA via surface-modified positively charged PEI-FA. Additionally, studies demonstrate that co-deliver miRNA-21 and IL-4, utilizing their complimentary roles in suppressing inflammation and fostering resolution [62].

At present, in view of the anti-inflammatory effect of M1-type macrophages induced to repolarize into M2-type in the process of RA, human serum albumin (HSA) has been used to co-deliver DEX and plasmid DNA (pDNA) expressing anti-inflammatory factors [63]. Close to this, the same two drugs were delivered using the cell membrane of M2-type macrophages as a carrier [64]. The synergy process is identical as well. In a similar vein, antioxidants and TNF-α siRNA can both be engineered to surround gold nanoparticles and administered simultaneously [65]. This strength allows nano-based co-delivery systems to be more adaptable when treating a variety of situations where the targets of nucleic acid medications are not identical.

#### 3.1.3. Co-Delivery with Enzyme-like Agents

Currently, the enzyme is used more and more widely in medicine. For instance, in vivo findings demonstrate that MTX and superoxide dismutase (SOD) delivery in combination are superior than monotherapy [66]. Enzyme-like agents generally refer to a group of molecules with specific enzymatic activities used to improve disease conditions. Metal-organic frames (MOF) have been developing very rapidly in recent years, especially in the field of biologics. This coordination polymer has a three-dimensional pore structure, generally with metal ions as the connection point and organic ligands supported to form a spatial 3D extension. MOFs are typically created to be enzyme active and further catalyze the breakdown of local hydrogen peroxide for anti-inflammatory purposes [67] because of the presence of metal ions. It has the most promise in the direction of RA therapy due to its high drug loading capacity and enzyme-like characteristics. Given the foregoing, it is possible to increase pharmacological inhibition or free radical scavenging by co-delivering enzyme-like molecules and other types of medications.

Pan et al. constructed MOFs formed by the coordination of monoacetate and zirconium atoms, and Au NPs were modified on the surface to improve photocatalytic H_2_ production efficiency [68]. This strategy demonstrated good therapeutic efficacy within 25 days by reducing reactive oxygen species (ROS) levels through H_2_ in combination with PTT therapy to kill target cells. It has also been studied that gold nanoparticles are encapsulated inside dendritic macromolecules to achieve inflammation inhibition by adsorbed nucleic acid drugs [65]. In addition, nanoparticles containing ligated superoxide dismutase and catalase enzymatic activity were created using mesoporous silica nanoparticles (MSNs) as a template [69]. MSN use would not be as straightforward as drug loading. Instead, the transition metal is doped using the sesquisiloxane backbone, and the enzymatic activity of the transition metal to scavenge ROS then controls the repolarization of macrophages. The same research team also made 2D MOFs [67], and the mechanism for treating RA is much the same. The experiments anchored two nanozymes, manganese ferrite, and cerium dioxide on a carrier that increased the oxygen production rate and alleviated the hypoxic environment at the site of joint inflammation [12]. From these, the co-delivery of agents, with enzymatic activity and other drugs, constitutes a cascade reaction or triggers each other’s pharmacological effects to achieve synergy more often.

High affinity and specificity are two of the noteworthy qualities of enzyme-like molecules as medications, and the other is that the products are typically metabolites in the biological self-reaction with less severe side effects. Nevertheless, the most criticized aspect of metal-derived nanoparticles is their intra-biological metabolism. Various studies are already trying to deal with this problem.

### 3.2. Co-Delivery for Overcoming Drug Limitation

Various types of drugs have different strengths and weaknesses. The optimization of nano-based co-delivery systems can enhance the advantages and improve the disadvantages of the drugs. As we all know, nanocarriers are limited in their drug loading capacity when only one drug is delivered due to intermolecular interactions between drugs [70]. Due to the low drug loading capacity, single drug loading also limits the efficacy of drug delivery. An important issue that needs to be resolved is the side effect. With higher doses, this frequently causes secondary toxicities to develop gradually. Or the drug may be released too quickly making administration problematic. Hence, several kinds of researches have been focused on these three aspects to explore co-delivery methods to overcome drug limitations.

#### 3.2.1. Enhance Drug Loading

The amount of drug that is carried by nanoparticles varies on the drug itself, as well as the size, shape, and nature of the particles. The large drug loading capacity that is challenging to attain when encapsulated in a carrier limits the potency and efficacy of many chemical medications, despite the fact that these drugs are efficacious in and of themselves. PLGA-made nanoparticles were used in research to contain MTX alone [71]. Despite the fact that over the course of two weeks there was a noticeable reduction in the arthritic process, based on paw volume and inflammatory factor expression levels, their drug loading and encapsulation rates were not noticeably higher. When MTX and gold nanoparticles were administered together, the drug loading was increased to 11.04% [72]. This includes another commonly used drug, DEX, which has a drug loading of only 7.5% when delivered alone [73]. In contrast, DEX and oligodeoxynucleotides (ODNs), gold nanorods (GNRs) were co-delivered with drug loading up to 35.63% [74]. Of course, there are many other studies that have identified many drugs acting on each other to improve drug loading. For instance, drug loading is significantly enhanced when pDNA encoding IL-10 and BSP are co-loaded into extracellular vesicles [64]. Celecoxib was adsorbed on boron nitride nanotubes to increase drug loading and enhance its anti-inflammatory effects [75]. All of these proved that co-delivery slightly improves the ability of the nanoparticles to carry the drug. There are many specific reasons why drug loading capacity is enhanced. Whether it is the electrostatic interaction between drugs or the fact that stable covalent bonds will be formed, these are the advantages that co-delivery can bring.

#### 3.2.2. Reduce Drug Side Effects

Any drug has side effects. It is well known that the long-term use of non-selective NSAIDs may lead to gastrointestinal abnormalities, such as perforation, ulceration, and bleeding, resulting in considerable mortality; side effects of MTX mainly include oral ulcers, cirrhosis, hepatitis, interstitial pneumonia, and hematocrit; toxic products of long-term use of GCs are related to cumulative dose. Many studies also focus on lessening the negative effects of medications. Co-delivery may be a great method to reduce adverse effects and potentially boost positive treatment outcomes at the same time, according to data from various research. The co-delivery of MTX and teriflunomide (TEF) reduced hepatotoxicity by 29% [76]. In some research, hepatorenal toxicity was assessed by evaluating renal and hepatic functional parameters after MTX was administered in combination with nimbolide. The similar results were found that the anti-inflammatory and antioxidant effects significantly reduced the hepatorenal toxicity produced by MTX, compared to monotherapy. Nano drugs that covalently bind MTX can also offer the potential for the selective accumulation of potent hepatotoxic drugs in rheumatic joints and with limited liver exposure [77]. How is a covalent modification not a co-delivery in another way? There is a study using liposomes to load paeoniflorin [78]. However, the experimental data exhibited some drug toxicity. The interference of the co-delivery method makes it different. The synergistic pharmacodynamics of paeoniflorin and LEF have been shown to enhance clearance and reduce liver injury [79]. As we all know, purified quercetin inhibits the action of adenosine deaminase (ADA), a critical inflammatory enzyme that increases joint stiffness and pain in RA [51]. When co-delivered with MTX, it enhanced the anti-inflammatory efficacy of the drug, increased antioxidant enzymes, and reduced oxidative stress to decrease its toxic effects. That means active substances, including those purified from medicinal plants, can also attenuate neurotoxicity through co-delivery with specific molecules. Instead of increasing side effects, the combination of two appropriate drugs for RA has been shown to have unexpected benefits. Reducing drug side effects through co-delivery may be a practical solution.

#### 3.2.3. Prolong the Duration of the Drug’s Effect

As we all know, the common problem of all nanoparticles is that they are prone to sudden release. Many studies have attempted to wrap layers of different size dosage forms to achieve slow release. This is due to the nature of the materials and matrices used to prepare nanoparticles, which are susceptible to degradation in a physiological environment and are in fact connected to the drug molecules inside. The cumulative release of PLGA polymer nanoparticles alone when encapsulated with MTX reached 60% at six hours [71]. According to available data, when MTX and transforming growth factor β1 (TGF β1) were co-loaded into chitosan complex hydrogels, especially when the amount of MTX was appropriate, the release of MTX was only 50% and TGF was only 45% for 60 days [80]. The hydro-gel possesses cross-linking, but it also features a dynamic equilibrium between the drug molecules brought on by hydrogen bonding, which significantly extends the time of drug release. It prolongs drug release much better than delivering MTX alone. Furthermore, nanoparticle hydrogels encapsulating LEF and DEX exhibited sustained release profiles of up to 58 and 17 days [56]. Naproxen and sulfapyridine demonstrated sustained release effects over eight hours as delivered using hydrogels [81]. Cyanidin, a natural alkaloid, was found to form a co-amorphous system with three NSAIDs, including indomethacin, naproxen, and sulindac, all of which provided sustained release data [82]. All of the above studies are direct evidence that co-delivery can prolong the duration of drug effects.

### 3.3. Co-Delivery for Imaging

With the advancement of biotechnology, therapy is no longer the only objective that researchers are working toward. In addition, patients anticipate imaging of RA areas. Therefore, more and more experiments are devoted to the exploration related to the integration of diagnosis and treatment. We tentatively categorize imaging into two types. One is the approach, which is frequently employed in animal research, of co-delivering fluorescent probes with drugs to observe drug distribution or targeting. The other is dynamic imaging, which can feed back different levels of imaging signals depending on the severity of the inflammation site. Either type of imaging has contributed in some way to the development of the integration of therapy and imaging.

Local imagery is typically easier to conceptualize. For drug tracking or in vivo imaging, fluorescent molecules and drugs are frequently co-delivered to the site of inflammation. The resulting isotopically labeled nanoparticles for the delivery of MTX are constructed [83,84]. In addition, it is also possible to achieve localization imaging by incorporating photosensitizers in the liposome preparation process [8]. Or, a gold half shell was created for the co-delivery of nanoparticles and MTX using the near-infrared (NIR) optical imaging capabilities of the gold [85]. These kinds of NIR local imaging can mainly observe the distribution of drugs in the body, which is aimed at the delivery effect and has little relationship with the degree of inflammation itself. That means the co-delivery system cannot overcomes the limitations of the drug or does not usually have a synergistic therapeutic effect. Simply encapsulating in the same nanoparticle and each having its own original effect is not the purpose of nanotechnology co-delivery, nor is it the desired results of scientific research.

Dynamic imaging is capable of demonstrating the severity of inflammation in the joint area. The first thing that comes to mind is the co-delivery of the contrast agent encapsulated in a therapeutic nano-based delivery system. For example, MnO_2_, one of the contrast agents for MRI, is co-delivered with MTX [86]. In order to act as both an agent and a contrast agent, He et al. co-delivered MTX, indocyanine green (ICG), and perfluoro pentane (PFP) enclosed inside PLGA nanoparticles [87]. PFP mediates photoacoustic imaging and dynamically tracks the severity of RA due to its low boiling point and ability to form bubbles in the phase that follows the ICG molecule converting light energy into heat energy. The target in this case is MTX. The primary reason for delivering the two drugs is so that one of them can cause the other to image. Likewise, the co-delivery of gas vesicles and MTX can achieve ultrasound imaging, even in real time for up to 5 days [8]. Additionally, the kind of inflammation-triggered disassembly nanoplatform cleverly accomplishes the dual purpose of therapeutic and dynamic imaging by utilizing down conversion nanoparticle (DCNPS) conjugated drugs with polymeric nanoparticles and fluorescence resonance energy transfer (FRET) technology [88]. Iron-containing nanoparticles and DCNPs are connected by disulfide bonds that break in response to inflammatory regions with high-glutathione concentrations. Afterward, the two nanoparticles are distanced from each other. The impact of FRET is lessened. Energy is discharged and then a signal is generated. Notably, this co-delivery technology treats patients by adsorbing nucleic acid medications, in addition to achieving dynamic imaging. In these cases, co-delivery imaging is no longer simply a co-loading of two molecules. Instead, energy transfer is cleverly used to enable the dynamic linkage between imaging and the inflammatory microenvironment. This not only provides more therapeutic benefits than conventional imaging agents, but also achieves control in the monitoring of the disease process in rheumatoid arthritis. Co-delivery imaging has also guided the application of co-delivery systems in other fields.

In general, co-delivery for imaging is a popular and challenging topic of research. If such strategies can achieve good results in the clinic, it will advance a big step forward in the integration of diagnosis and treatment in RA therapy.

### 3.4. Co-Delivery for Trigger Response

The so-called nano-based drug delivery system refers to the integration of drugs through materials and dosage forms. In addition, trigger-responsive formulations enable them to release drugs at the time and place needed in relatively safe doses to enhance efficacy further and reduce potential toxic side effects of drugs to meet the therapeutic needs of patients. Many endogenous molecules are present at the site of RA inflammation and inflammatory markers. For example, ROS, glutathione and the acidic microenvironment at the inflammation site can be considered targets for specific nanoparticles construction. This means that the designed drug-loaded nanoparticles can be released in response to these chemicals. In contrast, vehicles may also respond quickly and precisely to external stimuli, such as ultrasound or light. Currently, trigger-responsive nano-based co-delivery systems for achieving targeted and real-time release of drugs at inflammatory sites are a reality. In this section, we only review cases other than light-triggered responses. We will discuss phototherapy agents in more detail later.

#### 3.4.1. Chemicals of the Inflammatory Microenvironment

It is well established that the cellular microenvironment of RA sites is acidic. In order to generate a drug pH-triggered response, numerous acid-sensitive compounds can be used for the distribution alongside pharmaceuticals. When administering DEX and siRNA nanoparticles with acid-sensitive characteristics may typically achieve pH-responsive drug release [89]. However, this is more because of the nanoparticles in a weakly acidic environment for the lowering of explanatory drugs. Nanoparticles co-loaded with Prussian blue (PB) and indomethacin have a robust anti-inflammatory effect while also responding significantly to pH release [90]. Similar therapeutic results for RA were seen with PEG-modified PLGA polymer nanoparticles loaded with tetrandrine (Tet), particularly after hybridization with calcium carbonate NPs and encapsulation in microneedles, which improved PH response ability and enrichment at the focal site [91]. In addition, MTX and polydopamine (PDA) exhibited good pH sensitivity after co-delivery, and the results obtained showed that the cumulative release at pH 5.5 was twice as high as at pH 7.4 after 48 h [92]. Dong et al. co-delivered CaCO_3_ and BSO with MOF via an etching strategy [93]. CaCO_3_ dissolves in an acidic inflammatory environment releasing glutathione inhibitors, which in turn respond to treat RA. In some research, the generated ROS are used to kill activated macrophages and thus reduce the production of inflammatory factors. However, there are also responses that target autologous ROS production at the joint. The liposomes that co-deliver MTX and peroxidase liposomal membranes by enzymatically catalyzing the stress of generating oxygen [94]. In this case, the enzyme is no longer part of the synergy, but it acts as a drug release switch. The essential strategy is ROS response. Glutathione can reduce disulfide bonds. On this basis, Chen et al. immobilized linear β-(1,3)-glucan (BYG) and MTX on the surface and inside of methoxypoly(ethylene glycol) (mPEG), respectively, for co-delivery [95]. When the enriched GSH at the lesion site breaks the disulfide bond, the internal MTX is released to achieve responsive drug release. Such micelles have a drug loading capacity of up to 23.7%. A number of similar experiments are available. Above, the co-delivery of chemicals in response to the inflammatory microenvironment can indeed be achieved.

#### 3.4.2. External Stimulation

The combination of external stimulus–response and nano-based co-delivery systems is increasingly being used in research. Sound and light provided externally, for example, are more effective and better as drug release switches than the environmental response within the organism. So co-delivery of acoustic sensitizers and photosensitizers with drugs is the commonly used approach. The approximate principle of this kind of co-delivery systems is that the response to the external sound and light is used to disrupt the externally encapsulated nanoparticles, and the drug is released from inside. According to the literature, ultrasound-targeted microbubble disruption (UTMD) can enhance the ultrasonic cavitation effect and trigger the release of drugs from targeted nanocarriers in the synovial lumen, which is also an effective synergistic treatment strategy for RA [96,97]. Wu et al. developed an iRGD peptide-functionalized echo liposome that wraps MTX and ICG fluorescent probes by thin-film hydrophoresis [8]. It can also trigger MTX release in ultrasound. The efficacy of the treatment is unquestionable. The stability of the response is also confirmed in the related article. Of course, there is another way to make use of acoustic sensitizers. However, conventional acoustic sensitizers may not be as efficient as they should be. To improve that, Song et al. generated ROS based on zinc protoporphyrin sonosensitizer combined with heme oxygenase inhibitors to amplify oxidative stress and kill target cells under ultrasound conditions [98]. Acoustic sensitizer and nanozyme co-loading into nanoparticles can not only realize auditory response but can also synergistically enlarge sonodynamic therapy [99]. These studies have used the acoustic sensitizer itself as a drug to control the disease process in RA. Co-delivery on this basis also achieved enhanced therapeutic efficacy compared to conventional inefficiencies.

Without the use of auditory sensitizers, there are various ways to stimulate a response. When DEX and PFP administered together, for instance, the PFP acts as an initiator, causing the nanoparticles to “explode” and improve drug release [100]. Although ultrasound induces the response, it has also been employed in numerous additional investigations to complete photoacoustic imaging [101,102,103]. This is an added advantage that comes with ultrasound itself. In addition, it is the co-delivery that is the fundamental strength of the response triggered by external stimuli.

## 4. Nano-Based Co-Delivery System of Combination Phototherapy

**Figure 4:**
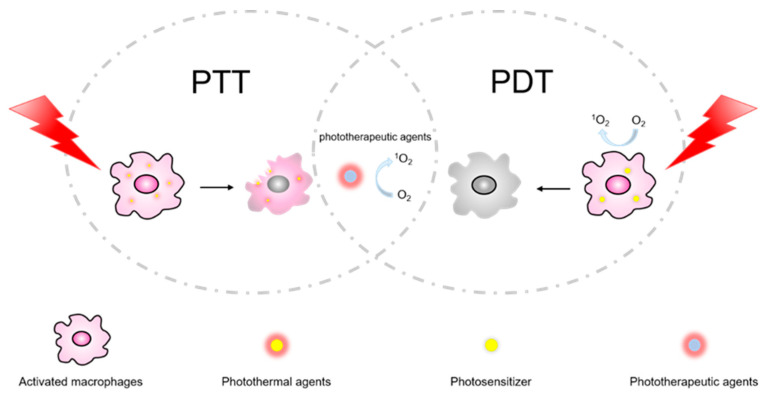
A brief overview of the different mechanisms of PDT and PTT therapies. They tend to rely on the co-delivery of photosensitizers and photothermal and phototherapeutic agents with both abilities.

We have reviewed many of the co-delivery models above. In fact, the most widely applied type is the co-delivery for trigger response, especially in combination with phototherapy. Photothermal therapy (PTT) and photodynamic therapy (PDT) are two different light-triggered therapies [104] (Figure 4). A combination of PTT and PDT may have better bone and cartilage preservation, less synovial invasion and a more substantial anti-inflammatory effect, which can effectively treat RA [105]. Photothermal agents and photosensitizers are important to light triggers for both therapies. Thus, they are often co-delivered with traditional agents (Table 1).

### 4.1. Co-Delivery of Photothermal Agents

Photothermal agents are a class of materials with good photothermal conversion ability. Light at a specific wavelength irradiates the photothermal agent, causing it to heat up and thus thermally ablate the cells. Different photothermal agents have other response conditions and trigger results. PDA is a good photothermal agent, since it is a polymer NIR-absorbing material with remarkable photothermal conversion efficiency and biocompatibility that can be totally destroyed in vivo. Pan et al. used the photothermal properties of PDA to design a nanoparticle to realize the PTT and H_2_ therapeutic group combined treatment of RA [68]. Pt NPs are evenly disseminated on the outer surface of the MOF, and the nanoparticle possesses a monodisperse octahedral crystal structure. They are based on MOF-loaded PDA and Perovskite quantum dots (QDs) to achieve the actualization of hydrogen thermal therapy. It has higher photocatalytic hydrogen production efficiency, while H_2_ is an antioxidant stress agent, capable of rapidly diffusing across cell membranes to reduce harmful ROS and protect cells from ROS damage. In addition, H_2_ can protect normal cells from hyperthermia damage [122]. According to the in vivo data, the co-delivery has an excellent photothermal effect. The temperature of the paw of the experimental group increased by 23.5 °C in the CIA mouse model, while the PBS group only increased by 4.5 °C. In addition, the paw joints of CIA mice in the experimental group were almost the same as those of normal mice. H&E staining also showed that the best group could significantly reduce the synovial tissue hyperplasia and cartilage erosion at the joint, and there was no obvious damage to the liver and kidney tissue. The mentioned results indicate that the co-delivery of multiple photothermal agents improves the efficiency of PTT while being an effective strategy for the treatment of RA.

Palladium blue, so named because of its blue tint, are hexagonal palladium nanosheets that were created in 2011 by Huang et al. [123]. The ultrathin palladium nanosheets exhibit excellent photothermal effects in vitro, as well as accurate and controllable surface plasmon resonance peaks in the near-infrared range. For PTT therapy, they can be used as photothermal agents. However, the poor biocompatibility of inorganic materials makes it challenging to use them alone. By targeting inflammatory cells and regulating MTX release with Pd particle and RGD peptide, Xu et al. designed and synthesized nanosheets for the treatment of RA taking into account the photo-thermal characteristics of palladium [110]. To improve their ability to target and get beyond the drawbacks of inorganic photothermal agents’ poor biocompatibility, RGD peptides can modify Pd NPs, an inorganic photothermal agent. In treating RA animals, the nanosheets demonstrated outstanding PTT efficacy, accumulating and significantly lowering inflammatory reactions, cartilage degradation and MTX toxicity in inflamed paws. This means that the nanosheets have good stability, photothermal conversion efficiency, and outstanding anti-inflammatory effects in vitro. This co-delivery of photothermal agent Pd and traditional drug MTX for the treatment of RA has many of the above advantages. So, it provides a potential approach for RA treatment.

One study synthesized a new multifunctional co-delivery system for the treatment of RA through a combination of PTT and antioxidant approaches [119]. They used a core-shell structured nanoparticle synthesis method to adsorb SeO_3_^2−^ onto ultra-small photothermally active Pd NPs with good photothermal activity. It was found that the Pd@Se-HA NPs had a more excellent photothermal effect compared with the control group. Under the influence of an 808 nm laser, the solution’s temperature rose to 44.5 °C. The photothermal effect of Pd@Se NPs and Pd@Se-HA NPs, however, may be less than that of Pd NPs alone, due to the shielding effect that Se NPs may have. As a result, RA can be treated with PTT and antioxidant combination therapy using Pd and Se NPs co-delivery systems. PEI was used as a stabilizer during the simple polymerization process that produced polypyrrole (PPy) NPs. The produced PPY-PEI NPs displayed a sizable NIR absorption, showing good photothermal performance and desirable photothermal stability [124]. Methylcellulose hydrogel co-loaded with strontium ranelate-laden (SrR) and PPY-PEI was used to treat RA [118]. From the examples above, it can be learned that photothermal agents and conventional drug co-delivery can not only respond to different wavelengths of light localized warming, but also do trigger drug release at the same time. Monotherapy is much less effective than combination therapy, both in terms of temperature control and factor interaction with the inflammatory microenvironment.

### 4.2. Co-Delivery of Photosensitizers

Photosensitizers are a class of compounds that facilitate chemical reactions by absorbing energy through light and then transferring it. They do not participate in the response and can even return to their initial state. In contrast, this type of reaction often has oxygen molecules involved in the biological reaction process. The applications have been quite widespread from the first generation of porphyrin-based photosensitizers to the second generation of PDT therapeutic drugs.

One study designed an extended O_2_/Ca^2+^-releasing phototherapeutic hydrogel (PO-Gel) system for joint injection treatment of RA [115]. By co-loading CaO_2_ and the photosensitizer Hematoporphyrin monomethyl Ether (HMME) into microspheres, this co-delivery system has a photo responsive capacity and a photo responsive capacity alleviates hypoxia at the joint site of PDT treatment and shows satisfactory therapeutic effects in a CIA rat model. In addition, IRDye-700DX is a light-activated chemical that binds antibodies and functions as a photosensitizer in PDT. A new strategy to trigger PDT for RA was developed and designed [116]. They coupled the photosensitizer IRDye-700DX with antibody via DTPA chelator for co-delivery. The same group further refined the above strategy by loading photosensitizers into liposomes to develop a new PDT therapy targeting macrophages [125]. Liposomes preferentially target photosensitizers to macrophages at joint inflammation, increasing the uptake of inflammatory cells and ROS cytotoxic effects. The data demonstrate that 700 DX-Liposomes are light dose-dependent triggers of PDT-induced cell death. In the CIA mouse model, arthritis scores in treated mice were significantly lower than in PBS controls during the first three days. There still was no significant difference in arthritis scores after the fifth day of treatment, showing a limited but transient effect, which is a weakness of most photosensitizers.

### 4.3. Co-Delivery of Phototherapeutic Agents for Combination Phototherapy

Besides some of the aforementioned photothermal and photosensitizers, there are some chemicals that have the capacity to generate both ROS and photothermal conversion. Pan et al. reported a novel multifunctional thermally responsive hydrogel system based on black phosphorus nanosheets [117]. This system treats RA synergistically with PTT and PDT. When irradiated with NIR, BPNs efficiently convert light energy into heat energy to produce ROS to destroy abnormal macrophages. The phosphorus contained in them is also an essential element for bone production. This co-delivery system has a therapeutic impact at the cellular level and partially restores the cartilage tissue that has been injured.

When it comes to chemicals with dual photo responsive functions, nanozymes are mandatory to be mentioned. The advantage of gold nanoparticles is related to the fact that by changing their shape, size, and composition, their optical properties can be tuned from the visible spectrum to the NIR spectrum. Gold nanorods, nanoshells, nanocages, and nanospheres are prominent phototherapeutic agents because of their strong light absorption in the visible and NIR spectra. The dual function makes phototherapeutic the first-line choice for nano-based co-delivery systems. For case in point, gold nanoparticles are used more frequently in the formulation field. One piece of work was devoted to synthesizing of a nanogold-core multifunctional dendrimer loaded with MTX to treat RA [112]. For the simultaneous delivery of MTX, the NIR bioactive agent IR780, and Au, they created a multifunctional platform. The results showed that the 808 nm laser is capable of producing photothermal effects and PDT at the site of inflammation, using their combined effects to boost RA effectiveness. For PTT, PDT, and targeted drug delivery for RA, Huang et al. have described a triple treatment based on octahedral copper sulfide shells and gold nanorod cores [120]. Under laser irradiation, local surface electromagnetic field coupling improves the electromagnetic field of Au cores and the light absorption of CuS shells to enhance the photothermal effect. This octahedral hollow can be loaded with MTX to achieve the co-delivery of drugs.

As early as 2013, Lee et al. synthesized a gold half-shell-based nanoparticle for the treatment of RA by combining it with the conventional chemotherapy MTX [106]. In CIA model mice, the combined use of this nanoparticle and gold enhanced the therapeutic effect. Later, MTX-laden nanospheres containing gold nanoparticles were developed successively for efficient targeting of chemiluminescence for the treatment of RA and with sound anti-inflammatory effects [107]. Kim et al. developed gold and iron nanoparticles for magnetically targeted chemoradiothermal treatment of RA [108]. Xue et al. encapsulated three anti-inflammatory agents, NF-κB decoy oligodeoxynucleotides, gold nanorods, and DEX, into folic acid-modified liposomes [121]. These gold substances exhibit photothermal conversion effects and photo-triggered ROS production effects when combined with NIR irradiation. Au NPs act together with PTT and PDT drove during NIR irradiation to produce local cytotoxicity and ROS toxicity for the treatment of RA. At the same time, heat can also be used to promote the release of conventional chemotherapeutics. Moreover, other phototherapeutic agents have shown favorable dual-dynamic therapy effects. A CuS NP based on copper nanoparticles and a novel l-cysteine-assisted synthesis was used as a novel modality for the treatment of RA [105]. In which copper stimulates chondrogenesis and osteogenesis while also acting as a photothermal and photosensitizer. Excellent photothermal and photodynamic effects, as well as strong biocompatibility, have been demonstrated in in vitro studies. Both metal particles and nanoparticles with composite structures are capable of producing singlet oxygen when exposed to light. This process turns light energy into heat, which is then used to destroy active macrophages that are producing inflammatory factors by using chemicals and temperature. The co-delivery of phototherapeutic agents with traditional chemical medications has the greatest potential for utilization.

## 5. Conclusions

In this review, we summarize the different effects of traditional co-delivery methods for RA. It is now typically used for co-treatment, overcoming drug limitations, imaging of inflammatory sites, and triggering responses. Different small molecules, nucleic acid drugs, and enzyme-like agents designed for co-delivery often have the ability to achieve the desired good results. However, while they offer improved treatment efficacy, compared to monotherapy, they have some limitations, such as possible interactions between different molecules of co-delivery or difficulties in controlling the sequence of release resulting in optimal RA treatment. In addition, this paper describes therapeutic strategies for nano-based co-delivery systems in combination with phototherapy, such as encapsulation with photosensitizers, photothermal agents, or bifunctional phototherapeutic agents, which provide conventional co-delivery with light-responsive or light-triggered drug release and have promising applications. The co-delivery system can be advantageous not only in the treatment of inflammation. It has considerable prospects in the treatment of neurodegenerative diseases or in the delivery to tumor sites to inhibit their growth. We believe that researchers can focus on co-delivery systems that provide more sensitive controversy and explore the multiple possibilities of action between co-loaded molecules in the next step. Therefore, nano-based co-delivery systems are expected to be future therapeutic strategies for treating RA in the future.

## Figures and Tables

**Table 1:** Co-delivery of phototherapy utilizing nanocarriers.

Formulation	Drugs Molecule	Phototherapeutic	Mechanism	Year	Ref
PLGA NPs	Methotrexate	Au half-shell	PTT	2012	[106]
PLGA NPs	Methotrexate	Au NPs	PTT	2015	[107]
PLGA NPs	Methotrexate	Au half-shells	PTT	2015	[108]
Nanowhiskers	TiO_2_	TSPP	PDT	2015	[109]
Nanosheets	Methotrexate	Pd nanosheets	PTT	2018	[110]
Metallic NPs	L-Cys	CuS NPs	PTT&PDT	2018	[105]
PLGA NPs	Resveratrol	QRu NPs	PTT	2019	[111]
Metallic NPs	Methotrexate	IR780&Au NPs	PTT&PDT	2019	[112]
MOFs	Doxorubicin	CuS NPs&RuII	PTT&PDT	2019	[113]
Liposomes	Dexamethasone	Gold nanorods	PTT	2020	[74]
PLGA NPs	Methotrexate	Au half-shells	PTT	2020	[85]
Liposomes	Tocilizumab	PBDTBBT	PTT	2020	[114]
Hydrogel	CaO_2_	HMME	PDT	2020	[115]
Micelles	Antibody 28H1	IRDye 700DX	PDT	2020	[116]
Hydrogel	Patelet rich plasma	Phosphorus nanosheets	PTT&PDT	2020	[117]
Hydrogel	Strontium Ranelate	Polypyrrole-PEI NPs	PTT	2021	[118]
Metallic NPs	Se NPs&Pd NPs	Pd NPs	PTT	2021	[119]
Hydrogel	Methotrexate	Fe_3_O_4_	PTT	2021	[80]
Metallic NPs	Methotrexate	Gold nanorods&CuS	PTT&PDT	2021	[120]
Mesoporous silica NPs	Tirapazamine	PCPDTBT	PTT&PDT	2021	[61]
MOFs	Pt NPs	Au NPs	PTT	2022	[68]
Membrane vesicles	Indomethacin	PB NPs	PTT	2022	[90]
Membrane vesicles	Schisanlactone E	PB NPs	PTT	2022	[121]

## Data Availability

Not applicable.
